# Soil nutrient limitation and natural enemies promote the establishment of alien species in native communities

**DOI:** 10.1002/ece3.10853

**Published:** 2024-01-22

**Authors:** Yu‐Han Xu, Yu‐Jian Guo, Yan‐Feng Bai, Yuan‐Yuan Liu, Yong‐Jian Wang

**Affiliations:** ^1^ College of Horticulture and Forestry Sciences Huazhong Agricultural University Wuhan China; ^2^ Research Institute of Forestry Chinese Academy of Forestry Beijing China

**Keywords:** natural enemies' suppression, nutrient addition, nutrient‐poor, plant invasion, select effect

## Abstract

The invasion of alien plant species threatens the composition and diversity of native communities. However, the invasiveness of alien plants and the resilience of native communities are dependent on the interactions between biotic and abiotic factors, such as natural enemies and nutrient availability. In our study, we simulated the invasion of nine invasive plant species into native plant communities using two levels of nutrient availability and suppression of natural enemies. We evaluated the effect of biotic and abiotic factors on the response of alien target species and the resistance of native communities to invasion. The results showed that the presence of enemies (enemy release) increased the biomass proportion of alien plants while decreasing that of native communities in the absence of nutrient addition. Furthermore, we also found that the negative effect of enemy suppression on the evenness of the native community and the root‐to‐shoot ratio of alien target species was greatest under nutrient addition. Therefore, nutrient‐poor and natural enemies might promote the invasive success of alien species in native communities, whereas nutrient addition and enemy suppression can better enhance the resistance of native plant communities to invasion.

## Graphical abstract

Nutrient‐poor and natural enemies might promote the invasive success of alien species in native community, whereas nutrient addition and enemy suppression can better enhance the resistance of native plant communities to invasion.

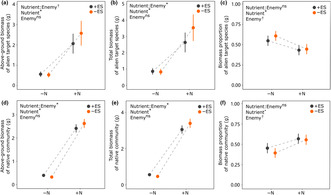

## 1 INTRODUCTION

Due to the increasing influence of human activities (Meyerson & Mooney, 2007; Turbelin et al., 2017), alien species continue to proliferate worldwide, potentially resulting in significant negative ramifications on ecosystems and human welfare (Kumschick et al., 2015; Paini et al., 2016). The invasion success of alien species may depend on environmental conditions in the invaded range, including both abiotic (Liu et al., 2017; Wang et al., 2017; Wang, Bai, et al., 2016) and biotic factors (Huang et al., 2020; Jin et al., 2022; Shan et al., 2022). Among them, it is widely acknowledged that the availability of environmental resources plays significant roles in facilitating the successful invasion of alien species (the resource fluctuation hypothesis; Davis et al., 2000; Enders et al., 2020). Indeed, a meta‐analysis has demonstrated that exotic plants have a greater ability to utilize added nitrogen compared to native plants (Liu et al., 2017). However, it is important to note that there are studies that contradict this hypothesis, suggesting that increased nutrient availability may suppress the invasion of exotic plants (Li, Gao, et al., 2022;  Zhang, van Kleunen, et al., 2021). Therefore, additional research is needed to examine the impacts of resource availability and identify the underlying factors that influence the varied responses of invasive plants to changes in resource availability. Such studies are crucial for gaining a better understanding of the implications and potential outcomes associated with these dynamics.

While the resource fluctuation hypothesis is regarded as a fundamental principle in invasion ecology, its testing has been limited to study systems within the same trophic level. However, it is important to acknowledge that plant growth can be significantly regulated by other trophic levels, such as herbivores and fungal pathogens (Agrawal et al., 2005; Beckmann et al., 2016; Heard & Sax, 2013; Maron & Vila, 2001). According to the enemy release hypothesis (Keane & Crawley, 2002; Liu & Stiling, 2006; Mitchell & Power, 2003), invasive species are attacked less by specialist enemies within their invaded range, which allows them to allocate more resources to growth (Carrillo & Siemann, 2016; Hierro et al., 2022; Rotter & Holeski, 2018). Furthermore, increasing availability of environmental resources can lead to improved quality of plant leaf tissue and increased palatability (Cornelis & Delvaux, 2016; van der Waal et al., 2011), thereby attracting a greater number of enemies that feed on these plants (Endara & Coley, 2011; van Langevelde et al., 2008). Overfeeding by enemies on plants can induce compensatory growth in plants. This compensatory growth, which occurs after herbivory, can benefit the invasive species by promoting their growth and establishment (Garcia & Eubanks, 2019; Getman‐Pickering et al., 2021; Hawkes & Sullivan, 2001; Ramula et al., 2019). Based on the above, it can be speculated that at the community level, the coexistence of high resources and natural enemies may have a combined effect on promoting or inhibiting invasion.

Previous studies have primarily focused on testing the resource fluctuation hypothesis and the enemy release hypothesis for one or a limited number of invasive plant species. Many of these studies have artificially created growth environments that enable the invasive species to thrive independently or compete with a single native species (Liang et al., 2020; Wang et al., 2019; Zhang et al., 2022; Zhao et al., 2023). However, this may not accurately represent the actual environment in which invasive species invade, as real‐world invasion scenarios are often more complex and involve multiple interacting species. At the community level, invasive plants encounter a diverse array of native plants, leading to selection effects on the native community (Adomako et al., 2019; Emery & Gross, 2007; Li, Jia, et al., 2022; Sun & Roderick, 2019). This dynamic can potentially enhance the competitive advantage of native plants, allowing them to effectively utilize and occupy environmental resources. Consequently, this can suppress the invasion of exotic plant species. Support for this hypothesis is derived from comparisons of traits and resource allocation between invasive and native communities (Dawson et al., 2012; Wang, Liu, et al., 2022; Wilsey & Polley, 2002). For instance, an increase in community evenness has been shown to impede the invasion of exotic plants (Tracy & Sanderson, 2004; Liu et al., 2019), while a low root‐to‐shoot ratio can facilitate the efficient utilization of resources by these invasive species, thereby augmenting their invasiveness (Ni et al., 2018). Nevertheless, little is known about the specific effect of different nutrient levels on the resistance of native ecosystems to invasion by alien plants.

To test the effects of nutrient availability and herbivores on the invasion of alien plant species in resident communities, a multispecies experiment was conducted. The experiment consisted of four combinations involving two levels of nutrient availability (low and high) and two treatments for natural enemy suppression (with and without suppression of natural enemies). We established a stable native community and transplanted each of nine alien invasive plant species as a target species. The study aimed to address the following questions: (i) Which levels of nutrients and natural enemies can promote the invasive success of alien species in native community? (ii) On the contrary, how can interactions between nutrient availability and natural enemies enhance the resistance of native plant communities to invasion?

## 2 MATERIALS AND METHODS

### 2.1 Species selection

The species for the experiment were selected from wetlands and grasslands in Nanshuihu National Wetland Park (N24°47′16″, E113°120′23″), located in Guangdong Province, China, based on a field investigation of the dominant alien invasive and native species in 2016. As target species for the experiment, nine alien clonal plant species were selected (i.e., *Hydrocotyle verticillata*, *Alternanthera philoxeroides*, *Sphagneticola trilobata*, *Erigeron annuus*, *Trifolium repens*, *Eleusine indica*, *Paspalum dilatatum*, *Ambrosia artemisiifolia*, *Amaranthus retroflexus*) which were dominant in the investigated habitats and co‐occurred in subtropical and tropical wetlands or grassland habitats in China (Ma, 2014, 2020; Ma & Li, 2018). To construct native communities for the experiment, six plant species commonly found and dominant in the wetlands and grasslands were chosen (Table S1). To balance the functional diversity in communities, the six native species belonged to the different functional groups (*Araliaceae*, *Amaranthaceae*, *Compositae*, *Lamiaceae*, *Oxalidaceae*, and *Rosaceae*). All the selected 15 herbaceous species co‐occur in the field (Table S1).

The species used in the experiment were collected from field sites in Guangdong Province, China. For alien clonal species, ramets were collected, while seeds were collected for other alien species and all native species (Table S1). The collected ramets were cultivated in a greenhouse at Huazhong Agricultural University (Wuhan, China) to produce enough new clonal fragments for the experiment. Additionally, the seeds were germinated in potting soil within the greenhouse to ensure the production of sufficient seedlings for the experiment. To account for variations in the time required for germination among the different species, the seeds were sown on different dates. This was done to ensure that all species were at similar developmental stages at the start of the experiment. The trays containing the seeds were placed in a greenhouse under natural light conditions, with a temperature ranging between 20 and 28°C. Each ramet and seedling used in the experiment had three leaves and some roots.

### 2.2 Experimental set‐up

The experiments were conducted in a greenhouse at Huazhong Agricultural University, located in Wuhan, China. The greenhouse was open on the sides to allow the entrance of insect herbivores and pollinators. To prevent the access to large animals (e.g., birds and mammals), we covered the sides with a sparse white nylon net (5 cm × 5 cm grids). For each of the nine target species, one ramet or seedling of an alien target species was transplanted in the center of each pot (24 cm long × 24 cm wide × 18 cm high). The six native plant species constitute a native community planted uniformly around the alien target species with a hexagonal design. One plant was planted in a pot per species. Each pot was filled with a 1:1 mixture of sand (0–0.5 mm) and yellow‐brown soil. The soil used for the experiment was collected from Shizishan Mountain in Wuhan, Hubei Province, China. The experimental design is shown in Figure S4.

In order to ensure that the nutrient content in the pots without nutrient addition treatment was at a lower level, the nutrient content of the mixed soil was determined. This was done to establish a baseline nutrient level for comparison with the low and high nutrient addition treatments in the experiment. The total N, total P, and total K of the mixed soil were mainly measured. The total N, P, and K content were 0.25 ± 0.03, 0.36 ± 0.04, and 18.24 ± 1.22 g/kg (mean ± SE, *n* = 10). To test the interactive effects of nutrient availability and enemy suppression on alien plant invasion into resident native communities, the 32 pots for each alien target species were assigned to two levels of nutrient availability (without vs. with nutrient) treatments, fully crossed with two levels of enemy suppression (without vs. with enemy suppression). In other words, per alien species, we had eight pots (i.e., replicates) in each of the four treatment combinations. To create different nutrient availability treatments, the soil was evenly mixed with one‐time application of 6 g water‐soluble fertilizer (20% N, 20% P_2_O_5_, 20% K_2_O, g/g, Peters Professional, Scotts, Geldermalsen, The Netherlands) as an added nutrient treatment (with nutrient). For the treatment with natural enemy suppression, the aboveground parts of the plants were sprayed with a broad‐spectrum insecticide (concentration: 2 mL/L; main ingredients: chlorpyrifos and fen valerate; The Dow Chemical Company, Midland, USA). Additionally, a solution containing a mixture of the same broad‐spectrum insecticide (concentration: 2 mL/L) and two broad‐spectrum fungicides was added to the soil every other week. The two fungicides used contained benzimidazole (1.5 g/L; Nufarm Limited, Contatti, Italy) and copper oxychloride (1.5 g/L; Dupont Agricultural Products, Washington DE, United States) as their main ingredients (Wang et al., 2019; Zheng et al., 2015). Additionally, we surrounded the area of the enemy suppression treatment with insect‐proof nets. For the control treatment without enemy suppression, the plants were sprayed with water instead of the broad‐spectrum insecticide and fungicide mixture. A total of 288 square pots (two levels of nutrient availability × two treatments for natural enemy suppression × nine target species × eight repetitions) were planted.

During the experiments, all plants were carefully watered to fulfill their growth requirements. And the pots were randomly positioned and reshuffled every 12 days to avoid the effects of possible environmental differences. The experiment was conducted from August to October 2015, lasting for 10 weeks in a greenhouse under natural sunlight. During the experiment, the mean temperature in the greenhouse was set to 27.7°C and the relative humidity to 70.5% (measured by Amprobe TR300, Amprobe, Everett, WA, USA). The light intensity in the greenhouse was 70% of that outside.

### 2.3 Plant harvest and measurements

At the end of the experiment, plants were harvested and separated into above‐ground and below‐ground parts of the target species and native community in each pot. Aboveground biomass refers to the total biomass of stems and leaves, while belowground biomass refers to the root biomass of plants. All biomass samples were dried at 65°C for 72 h and then weighed. Moreover, the biomass proportion was used as an indicator of plant growth of alien species in the whole community, and it was calculated from the proportion of total biomass of the alien target plant to total biomass of the whole community (alien target species plus sum of biomass of all native species). The root‐shoot ratio is calculated by dividing the belowground biomass by the aboveground biomass. In addition, to evaluate the evenness of the native community, the Shannon evenness index was calculated: *J*′ = *H*′/ln(*S*), where *H*′ is the Shannon–Wiener diversity index: *H*′ = −∑*p*_*i*_ln(*p*_*i*_), where *p*_*i*_ is the proportional biomass of each species and *S* is the number of species in the community (Kardol et al., 2010).

### 2.4 Statistical analyses

To test for differences in biomass production between target alien species and native communities in response to nutrient addition and enemy suppression, we fitted a linear mixed‐effects model in R 4.1.1 (R Core Team, 2021) using the lme function in the nlme package (Pinheiro et al., 2017, 2020). To improve normality and homoscedasticity of the residuals, above‐ground biomass production, biomass production, and root/shoot ratio of alien target species and the native communities were log‐transformed, biomass proportion of the alien target species and native communities in each pot was logit‐transformed, and evenness of native community was asin‐transformed prior to analyses. The fixed part of the model included nutrient addition (with vs. without nutrient), enemy suppression (with vs. without enemy), and all their interactions. To account for non‐independence of plants from the same species and non‐independence of species from the same genus, models included family and species (nested within genus) as random effects.

## 3 RESULTS

### 3.1 Biomass of alien target species and native community

**FIGURE 1:**
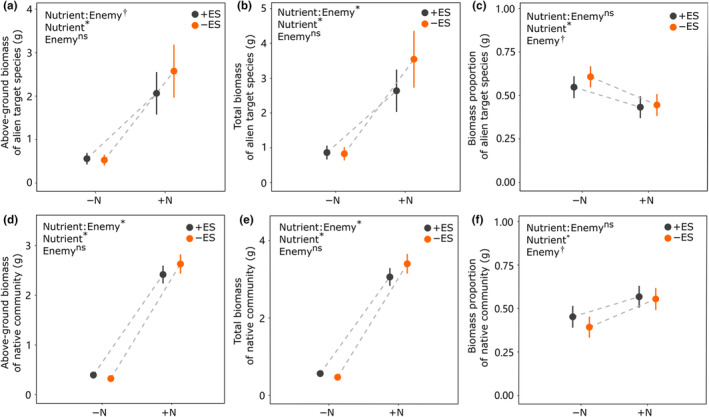
Effects of nutrient addition (without vs. with), enemy suppression (with vs. without), and their interaction on total biomass, above‐ground biomass, and biomass proportion for alien target species and native community. +ES = with enemy suppression, −ES = without enemy suppression, +N = with nutrient addition, −N = without nutrient addition. Significance levels: *p* < .05 are indicated with asterisks (*), .05 < *p* < .1 are indicated with daggers (^†^), *p* > .1 are indicated with “ns.”

Nutrient addition significantly increased total biomass production and above‐ground biomass of alien target plant species and native community, respectively (total biomass production and above‐ground biomass production‐alien species vs. native community: 257.6% (*p* < .000) vs. 527.7% (*p* < .000), and 320.0% (*p* < .000) vs. 604.3% (*p* < .000); Figure 1a,b,d,e; Table 1). However, nutrient addition significantly decreased biomass proportion of alien species in comparison to the increase of native community (alien species vs. native community: −13.6% (from 58.9% to 45.3%, *p* < .000) vs. 13.6% (from 41.1% to 54.7%, *p* < .000); Figure 1c,f; Table 2). The presence of enemies marginally significantly increased biomass proportion of alien plants and decreased that of native communities (alien target species vs. native community: 6.1% (from 49.6% to 52.6%, *p* = .069) vs. −6.1% (from 50.4% to 47.4%, *p* = .069); Figure 1c,f; Table 2). Especially, compared to the nutrient addition treatment, the presence of enemies increased the biomass proportion of alien plants by 10.9% (from 54.8% to 60.7%, *p* = .266) and decreased that of native communities by −13.1% (from 45.2% to 39.3%, *p* = .268) without nutrient addition (Figure 1c,f; Table 2), although these interactions were not significant.

**TABLE 1:** Linear mixed‐effect models testing the effects of the nutrient addition (without vs. with), enemy suppression (with vs. without) treatments, and their interactions on above‐ground biomass, below‐ground biomass, and total biomass of the alien target species and the native communities.^a^

Explanatory variables (fixed and random effects)		Below‐ground biomass of alien target species (natural‐log‐transformed)		Above‐ground biomass of alien target species (natural‐log‐transformed)		Total biomass of alien target species (natural‐log‐transformed)		Below‐ground biomass of native communities (natural‐log‐transformed)		Above‐ground biomass of native communities (natural‐log‐transformed)		Total biomass of native communities (natural‐log‐transformed)	
	Df	χ^2^	*p*	χ^2^	*p*	χ^2^	*p*	χ^2^	*p*	χ^2^	*p*	χ^2^	*p*
Nutrient addition (N)	1	33.060	**<.000**	218.447	**<.000**	171.388	**<.000**	209.594	**<.000**	356.079	**<.000**	322.452	**<.000**
Enemy suppression (ES)	1	4.704	**.030**	0.851	.851	1.980	.159	0.860	.354	1.034	.309	0.515	.473
N × ES	1	3.171	.075	3.285	.070	4.242	**.039**	5.548	**.019**	4.698	**.030**	4.576	**.032**
Random effects		SD		SD		SD		SD		SD		SD	
Family		0.100		0.000		0.143		<0.000		0.079		0.035	
Species		0.164		0.706		0.684		0.278		0.241		0.255	
Residual		0.161		0.630		0.666		0.664		0.411		0.408	
R^2^ of the model		Marginal	Conditional	Marginal	Conditional	Marginal	Conditional	Marginal	Conditional	Marginal	Conditional	Marginal	Conditional
		0.112	.633	0.368	.720	0.309	.671	0.518	.590	0.804	.858	0.785	.846

*Note*: Significant effects (*p* < .05) are in bold, while marginal significant effects (.05 < *p* < .1) are underlined.

^a^ Standard deviations for individual alien species random effects for the saturated model are found in Table S2.

**TABLE 2:** Linear mixed‐effect models testing the effects of the nutrient addition (without vs. with) and enemy suppression (with vs. without) treatments and their interactions on biomass proportion of the alien target species and the native communities and the evenness of native communities.^a^

Explanatory variables (fixed and random effects)		Biomass proportion of the alien target species (logit‐transformed)		Biomass proportion of the native communities (logit‐transformed)		The evenness of native communities (asin‐transformed)	
	Df	χ^2^	*p*	χ^2^	*p*	χ^2^	*p*
Nutrient addition (N)	1	27.023	**<.000**	27.041	**<.000**	110.936	**<.000**
Enemy suppression (ES)	1	3.301	.069	3.314	.069	4.844	**.028**
N × ES	1	1.236	.266	1.225	.268	3.022	.082
Random effects		SD		SD		SD	
Family		0.150		0.150		0.000	
Species		0.855		0.856		0.114	
Residual		0.670		0.670		0.224	
*R* ^2^ of the model		Marginal	Conditional	Marginal	Conditional	Marginal	Conditional
		0.088	.660	0.088	.660	0.311	.452

*Note*: Significant effects (*p* < .05) are in bold, while marginal significant effects (.05 < *p* < .1) are underlined.

^a^ Standard deviations for individual alien species random effects for the saturated model are found in Table S2.

The presence of an enemy increased above‐ground biomass and total biomass of alien plants and native communities under nutrient addition (24.7% vs. 8.8% and 34.3% vs. 11.1%; Figure 1b,e; Table 1), whereas the opposite was true without nutrient addition (−5.8% vs. −18.0% and −4.1% vs. −16.8%; Figure 1b,e; Table 1) (significant interaction of Nutrient × Enemy; *p* = .070 vs. *p* = .039 and *p* = .030 vs. *p* = .032). Therefore, the positive effect of enemy suppression (i.e., absence of enemy) was higher when without nutrient addition, and the effect turned over under nutrient addition (Figure 1b,e; Table 1; Table S3). From the results, nutrient addition promotes biomass compensation for enemies' feeding.

### 3.2 Effects on evenness of native communities

**FIGURE 2:**
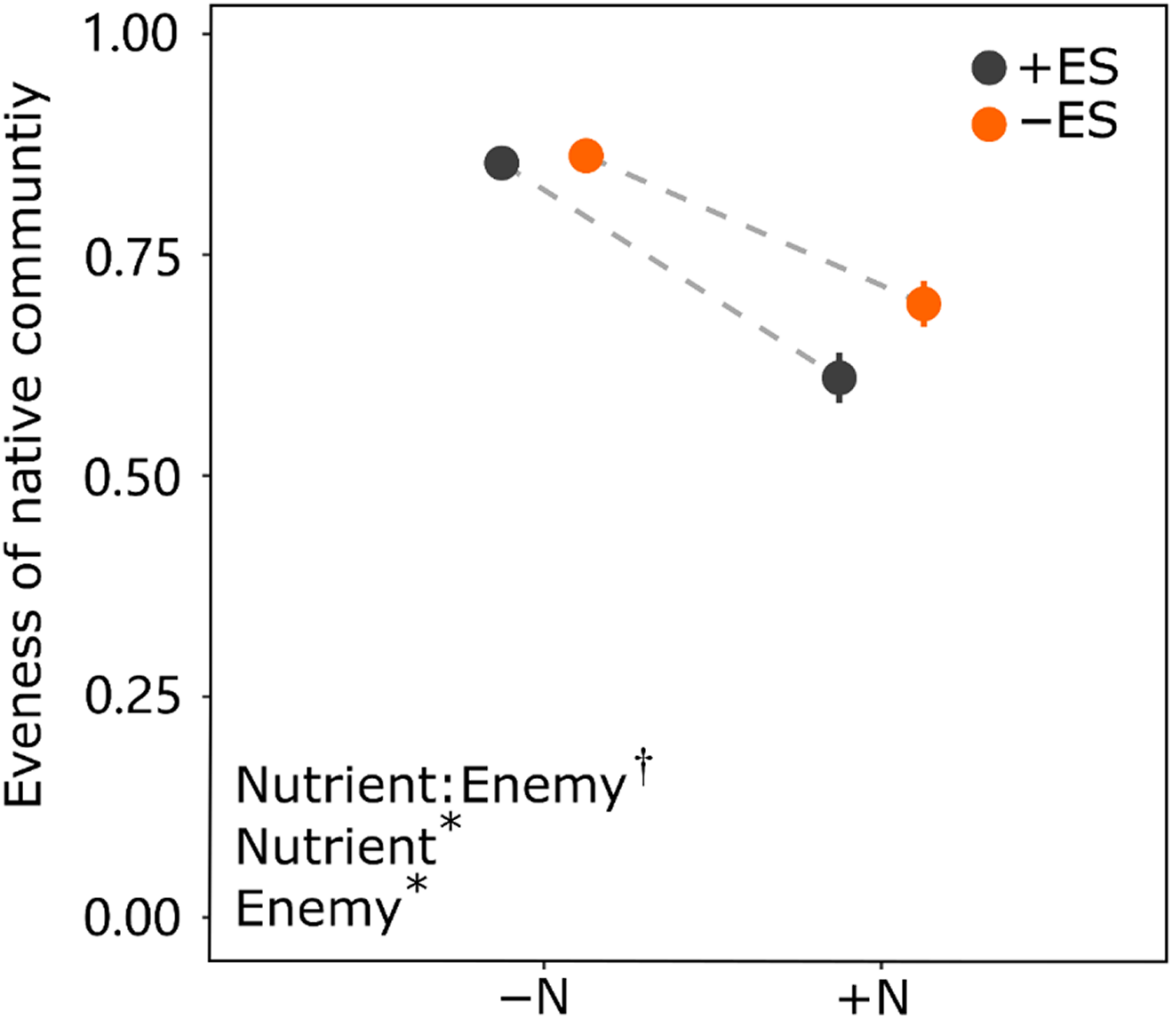
Effects of nutrient addition (without vs. with), enemy suppression (with vs. without), and their interaction on evenness for native community. +ES = with enemy suppression, −ES = without enemy suppression, +N = with nutrient addition, −N = without nutrient addition. Significance levels: *p* < .05 are indicated with asterisks (*), .05 < *p* < .1 are indicated with daggers (^†^), *p* > .1 are indicated with “ns.”

Nutrient addition and enemy suppression significantly decreased evenness of native community (−24.3% (*p* < .000) and −1.6% (*p* = .028); Figure 2; Table 2), whereas the negative effect of enemy suppression was maximal under nutrient addition (nutrient addition vs. without nutrient addition: −12.1% vs. −0.9%; *p* = .082; Figure 2; Table 2).

### 3.3 Effects on root‐shoot ratio of alien target species and native communities

**FIGURE 3:**
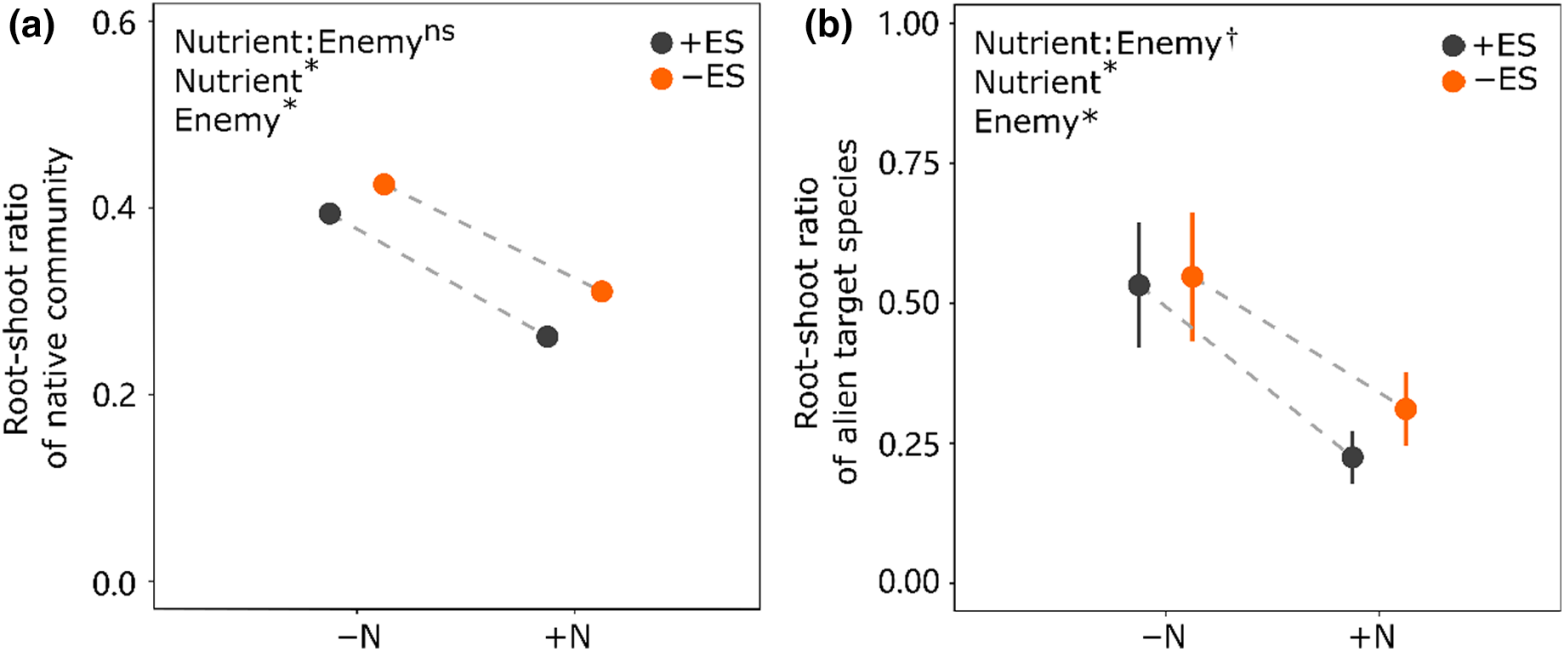
Effects of nutrient addition (without vs. with), enemy suppression (with vs. without), and their interaction on root‐shoot ratio for alien target species and native community. +ES = with enemy suppression, −ES = without enemy suppression, +N = with nutrient addition, −N = without nutrient addition. Significance levels: *p* < .05 are indicated with asterisks (*), .05 < *p* < .1 are indicated with daggers (^†^), *p* > .1 are indicated with “ns.”

Nutrient addition significantly decreased root‐shoot ratio of native communities and alien target species (−30.1% (*p* < .000) vs. −51.5% (*p* < .000); Figure 3; Table 3). The presence of enemies significantly increased root‐shoot ratio of native community and alien target species (2.1% (*p* = .001) vs. 1.5% (*p* = .025); Figure 3; Table 3). However, the negative effect of enemy suppression on alien target species was maximal when under nutrient addition (nutrient addition vs. without nutrient addition: −55.5% vs. −40.7%; Figure 3b; Table 3) (significant interaction of Nutrient × Enemy; *p* = .070). Most clonal target alien species (*A. philoxeroides*, *S. trilobata*, *T. repens*) showed similar pattern of root‐shoot ratio to those when all alien species were used (Figures S1 and S2).

**TABLE 3:** Linear mixed‐effect models testing the effects of the nutrient addition (without vs. with), enemy suppression (with vs. without) treatments, and their interactions on root‐shoot ratio of the alien target species and the native communities.^a^

Explanatory variables (fixed and random effects)		Root‐shoot ratio of the alien target species (natural‐log‐transformed)		Root‐shoot ratio of native communities (natural‐log‐transformed)	
	df	χ^2^	*p*	χ^2^	*p*
Nutrient addition (N)	1	58.636	**<.000**	81.903	**<.000**
Enemy suppression (ES)	1	5.027	**.025**	10.282	**.001**
N × ES	1	3.276	.070	1.582	.208
Random effects					
Family		0.000		0.000	
Species		0.637		0.000	
Residual		0.478		0.302	
R^2^ of the model		Marginal	Conditional	Marginal	Conditional
		0.186	.707	0.295	.295

*Note*: Significant effects (*p* < .05) are in bold, while marginal significant effects (.05 < *p* < .1) are underlined.

^a^ Standard deviations for individual alien species random effects for the saturated model are found in Table S2.

## 4 DISCUSSION

Our multispecies experiment showed that despite without nutrient addition and the presence of enemies (enemy release), there was a significant promotion in the absolute biomass of the alien target species. However, this promotion also facilitated the dominance of the alien target species within the native community. In other words, nutrient addition and the absence of enemies (i.e., enemy suppression) enhanced the competitive advantage of the native community. One of the reasons for this is that under nutrient addition conditions, enemy suppression had the greatest negative impact on the evenness of the native community and the root‐shoot ratio of the alien target species, thereby promoting the selection effect and competitive dominance of the native community. These findings imply that high nutrient and enemy suppression can better enhance the resistance of native communities to invasion of alien plants, and nutrient limitation and enemy release environments might promote invasive success of alien plants.

### 4.1 Effects on biomass production

The presence of enemies and without nutrient addition resulted in an increase in the dominance of alien plants within the community and a decrease in the dominance of the native community. This indicates that enemies had a slight positive impact on the performance of alien target species in the native community under nutrient‐deficient conditions (Figure 1). The reason for this result may be competitive advantage and compensation effects between native communities and invasive plants under different nutrient and herbivory environments. On the one hand, under conditions of limited resources, alien target species may have higher resource utilization efficiency and growth performance than native communities (Funk & Vitousek, 2007; Heberling & Fridley, 2013; Littschwager et al., 2010; Liu et al., 2019). On the other hand, those that originated in fertile habitats frequently have relatively higher resource requirements and utilization skills than those that originated in poor habitats, such as sandy soils or semi‐arid areas, which typically have lower resource requirements (Funk, 2013; Grassein et al., 2010; Grime, 1977; Wright et al., 2004). Most of the alien target species used in this experiment initially invaded barren and impoverished areas (Hyun et al., 2020; Khatun et al., 2010; Marks, 1983; Snaydon, 1962). This may explain the greater competitiveness of alien invasive species in low‐nutrient environments. In addition, in the absence of nutrient additions, alien target species in invasion areas lacking specialist enemies and the preference of generalist enemies can allocate more resources to their own growth compared to native communities facing both specialist and generalist enemies (Keane & Crawley, 2002; Meijer et al., 2016; Wang et al., 2019; Zhang et al., 2018). And native species in resident communities also face more enemies than invasive target species (i.e., co‐existing native herbivores) in invaded areas (Keane & Crawley, 2002; Zhang et al., 2020), which also results in greater compensatory effects. Therefore, enemy release will marginally reduce the invasion resistance of native communities and promote the invasion of alien plants without nutrient additions. Conversely, enemy suppression promote native community and alien target species biomass under nutrient addition, in line with previous findings that plants compensate for or tolerate enemies more when growing with nutrient supply (Gange, 2000; Meyer, 2000; Ramula et al., 2019; Wang et al., 2019; Zhong et al., 2021).

### 4.2 Effects on evenness of native communities

Our finding indicated that the negative impact of enemy suppression on the evenness of the native community was more pronounced under nutrient addition compared to without. Additionally, despite the decrease in evenness (Figure 2), the proportion of the native community increased under nutrient conditions (Figure 1f). As a result, the dominant species, *Glechoma longituba* produced significantly more biomass (Figure S1a,b), which marginally inhibited the invasion of alien target plants both above‐ and below‐ground (Figure 1a; Figure S3). Our results were consistent with the previous findings (Li, Gao, et al., 2022; Parepa et al., 2013; Roscher et al., 2016) that species with strong competitive abilities can better utilize available resources, leaving fewer resources for invasive species due to selection effects (Adomako et al., 2019; Li, Jia, et al., 2022; Van Ruijven & Berendse, 2003). As a result of nutrient addition, some key species in native communities with high competitiveness accumulate more biomass and occupy more resources, effectively inhibiting invader invasion.

### 4.3 Effects on root‐to‐shoot ratio

We observed that after nutrient availability increased and enemy suppression, there was a greater negative impact on the root‐shoot ratio of alien species compared to the native community (Figure 3). These findings are consistent with previous research indicating that there are differences in root and shoot allocation between invasive alien species and native species (Van Kleunen et al., 2010), with invasive species generally exhibiting lower root‐to‐shoot ratios compared to non‐invasive species when considering the same total biomass. (Schlaepfer et al., 2010). The negative effect of enemy suppression on the root‐to‐shoot ratio of alien target species, however, was most pronounced when nutrients were added. This outcome can be influenced by factors both above‐ and below‐ground. On the one hand, when using pesticides to suppress enemies, both the invasive and native communities are hardly damaged (as previously demonstrated in our lab by Wang et al., 2019). At this point, the increase in soil nutrient content triggers native community selection effects (Zhang, Gao, et al., 2021), enhancing the competitive advantage of the dominant species *Glechoma longituba*, which prioritizes resource allocation to its roots, thus occupying soil nutrient resources and suppressing the invasion of alien species. Without nutrient addition and with the presence of enemies, when invasive plants grow in a competitive environment, there is no significant difference in root‐to‐shoot ratio between native communities and invasive plants. This is mainly because the dominant species in the native community do not grow prominently without nutrients addition. In our greenhouse experiment, the main enemies are generalist herbivores, including the larvae of the striped stem borer and the cabbage looper (Wang, personal observation), which are likely to feed on both native and invasive plants (Wang et al., 2019). On the other hand, the soil biota has a positive effect on plant growth (Jin et al., 2022; Lussenhop & BassiriRad, 2005; Mehring & Levin, 2015; Partsch et al., 2006). However, a higher root‐to‐shoot ratio is related to the ability of plants to access below‐ground resources (nutrients and water) and is crucial for establishing ecosystems in competitive environments (Casper & Jackson, 1997; Ferguson et al., 2015). A larger root system in native communities and a more resilient below‐ground plant‐biota network formed with soil biota promote nutrient acquisition and biomass growth advantages, thus impeding the invasion of exotic plants.

### 4.4 Potential limitations

One caveat for our study is that because our native community only used a single fixed set of native plant species, random distinction possibilities of the diversity effect were not considered (Abernathy et al., 2016; Liao et al., 2015; Qin et al., 2020). However, our native community composition and diversity were designed based on the previous field investigation. Meanwhile, our study was conducted in a greenhouse and was rather short. Finally, we found that not all alien target species responded to the nutrient and enemy suppression treatments in the same way.

## 5 CONCLUSION

When invading new habitats, alien plants face complex environmental conditions where various abiotic factors and multiple trophic levels may impact their success. Our experimental findings suggest that nutrient‐poor conditions and the presence of natural enemies may contribute to the successful invasion of alien species in native communities. Conversely, nutrient addition and enemy suppression can enhance the resistance of native plant communities against invasion. However, the interactive effect between nutrient addition and enemies' suppression was not significant in our study, possibly due to the limited number of species used and the homogeneous community composition. This implies that as eutrophication increases, native communities may develop stronger resistance to invasiveness, with natural enemies potentially playing a crucial role in this process.

## AUTHOR CONTRIBUTIONS

**Yu‐Han Xu:** Data curation (lead); formal analysis (lead); methodology (lead); writing – original draft (lead). **Yu‐Jian Guo:** Data curation (supporting). **Yan‐Feng Bai:** Writing – review and editing (equal). **Yuan‐Yuan Liu:** Investigation (lead). **Yong‐Jian Wang:** Conceptualization (lead); funding acquisition (supporting); resources (supporting); validation (lead); writing – review and editing (equal).

## CONFLICT OF INTEREST STATEMENT

The authors declare no conflict of interest.

## Supporting information


Data S1

## Data Availability

The experimental data and code are given in Supporting Information S1.
