# Supplementary material for: Soil nutrient limitation and natural enemies promote the establishment of alien species in native communities
Source: Ecol Evol. 2024 Jan 22;14(1):e10853. doi: 10.1002/ece3.10853 (PMC10803180; doi:10.1002/ece3.10853)

# Supporting information

# Extended data Table S1 Details of the study species used in the experiment. -

| Species | Genus | Family | Status | Life history |
| --- | --- | --- | --- | --- |
| *Hydrocotyle sibthorpioides* Lam. | Hydrocotyle | Araliaceae | Native | Perennial |
| *Alternanthera sessilis* (L.) R.Br. ex-DC*.* | Alternanthera | Amaranthaceae | Native | Perennial |
| *Sphagneticola calendulacea* (L.) Pruski | Sphagneticola | Compositae | Native | Perennial |
| *Glechoma longituba* (Nakai) Kuprian. | Glechoma | Lamiaceae | Native | Perennial |
| *Oxalis corniculata* L. | Oxalis | Oxalidaceae | Native | Perennial |
| *Duchesnea indica* (Jacks.) Focke | Duchesnea | Rosaceae | Native | Perennial |
| *Hydrocotyle verticillata* Thunb*.* | Hydrocotyle | Araliaceae | Alien | Perennial |
| *Alternanthera philoxeroides* (Mart.) Griseb. | Alternanthera | Amaranthaceae | Alien | Perennial |
| *Sphagneticola trilobata* (L.) Pruski | Sphagneticola | Compositae | Alien | Perennial |
| *Erigeron annuus* (L.) Pers. | Erigeron | Compositae | Alien | Annual |
| *Trifolium repens* L. | Trifolium | Fabaceae | Alien | Perennial |
| *Eleusine indica* Gaertn. | Eleusine | Poaceae | Alien* | Annual |
| *Paspalum dilatatum* Poir. | Paspalum | Poaceae | Alien | Perennial |
| *Ambrosia artemisiifolia* L. | *Ambrosia* | Compositae | Alien | Annual |
| *Amaranthus retroflexus* L. | Amaranthus | Amaranthus | Alien | Annual |

Status and habitat information is based on the Flora of China (www.efloras.org), on the Scientific Database of China Plant Species (DCP) (http://www.plants.csdb.cn/eflora) and on additional references as cited.

* The original center of the species is East Africa (www.cabidigitallibrary.org).

# Extended data Table S2 Standard deviation of individual species random effects for the metrics analyzed with Gaussian error distribution models. The standard deviations given refer to the first species respectively. For each species, these should be multiplied by the multiplication factors. The names of the species in the table are abbreviated using the first letter of the genus and species epithet.

| **Metric** | **Multiplication factor standard deviation** | | | | | | | | |
| --- | --- | --- | --- | --- | --- | --- | --- | --- | --- |
|  | **AP** | **AR** | **ST** | **AA** | **EA** | **TR** | **EI** | **PD** | **HV** |
| Below-ground biomass of alien target species | 1.000 | 1.786 | 0.925 | 1.589 | 2.317 | 1.045 | 1.193 | 1.476 | 0.655 |
| Above-ground biomass of alien target species | - | - | - | - | - | - | - | - | - |
| Total biomass of alien target species | - | - | - | - | - | - | - | - | - |
| Below-ground biomass of native communities | - | - | - | - | - | - | - | - | - |
| Above-ground biomass of native communities | 1.000 | 1.803 | 1.275 | 1.146 | 1.916 | 1.125 | 1.071 | 1.278 | 1.389 |
| Total biomass of native communities | 1.000 | 1.849 | 1.340 | 1.277 | 1.789 | 1.194 | 1.133 | 1.307 | 1.493 |
| Biomass proportion of the alien target species | 1.000 | 1.861 | 0.963 | 0.772 | 1.533 | 1.345 | 1.420 | 1.114 | 1.296 |
| Biomass proportion of native communities | 1.000 | 1.864 | 0.964 | 0.773 | 1.531 | 1.346 | 1.419 | 1.114 | 1.296 |
| Root-shoot ratio of the alien target species | 1.000 | 1.815 | 0.919 | 2.105 | 2.525 | 1.867 | 1.818 | 1.391 | 0.890 |
| Root-shoot ratio of native communities | - | - | - | - | - | - | - | - | - |
| The evenness of native communities | - | - | - | - | - | - | - | - | - |

# Extended data Table S3 The result of the general linear model testing the effects of different enemy suppression treatments on above-ground biomass and total biomass of the alien target species and the native communities. Significant effects (*P* < 0.05) are in bold, while marginal significant effects (0.05<*P* < 0.1) are underlined.

| **Fixed effect** |  | **Aboveground biomass of alien target species** | | **Total biomass of alien target species** | | **Aboveground biomass of native communities** | | **Total biomass of native communities** | |
| --- | --- | --- | --- | --- | --- | --- | --- | --- | --- |
|  | **DF** | **F** | ***P*** | **F** | ***P*** | **F** | ***P*** | **F** | ***P*** |
| Nutrient addition | 1 | 7.058 | **0.009** | 11.290 | **0.001** | 1.929 | 0.167 | 3.914 | 0.050 |
| Without nutrient addition | 1 | 0.225 | 0.636 | 0.078 | 0.780 | 29.37 | **<0.000** | 25.57 | **<0.000** |

# Fig. S1 Relative nutrient addition (A) and enemy suppression (B) responses for total biomass, above-ground biomass, below-ground biomass, root: shoot (R: S) ratios, and biomass proportion for each native and alien target species. Bars represent means ±SE. Asterisks above bars for each species indicate a significant enemy suppression and nutrient addition response from t-tests comparing trait values: *, 0.05＞*P* ＞0.1; **, *P* < 0.05; ***, *P* < 0.01.


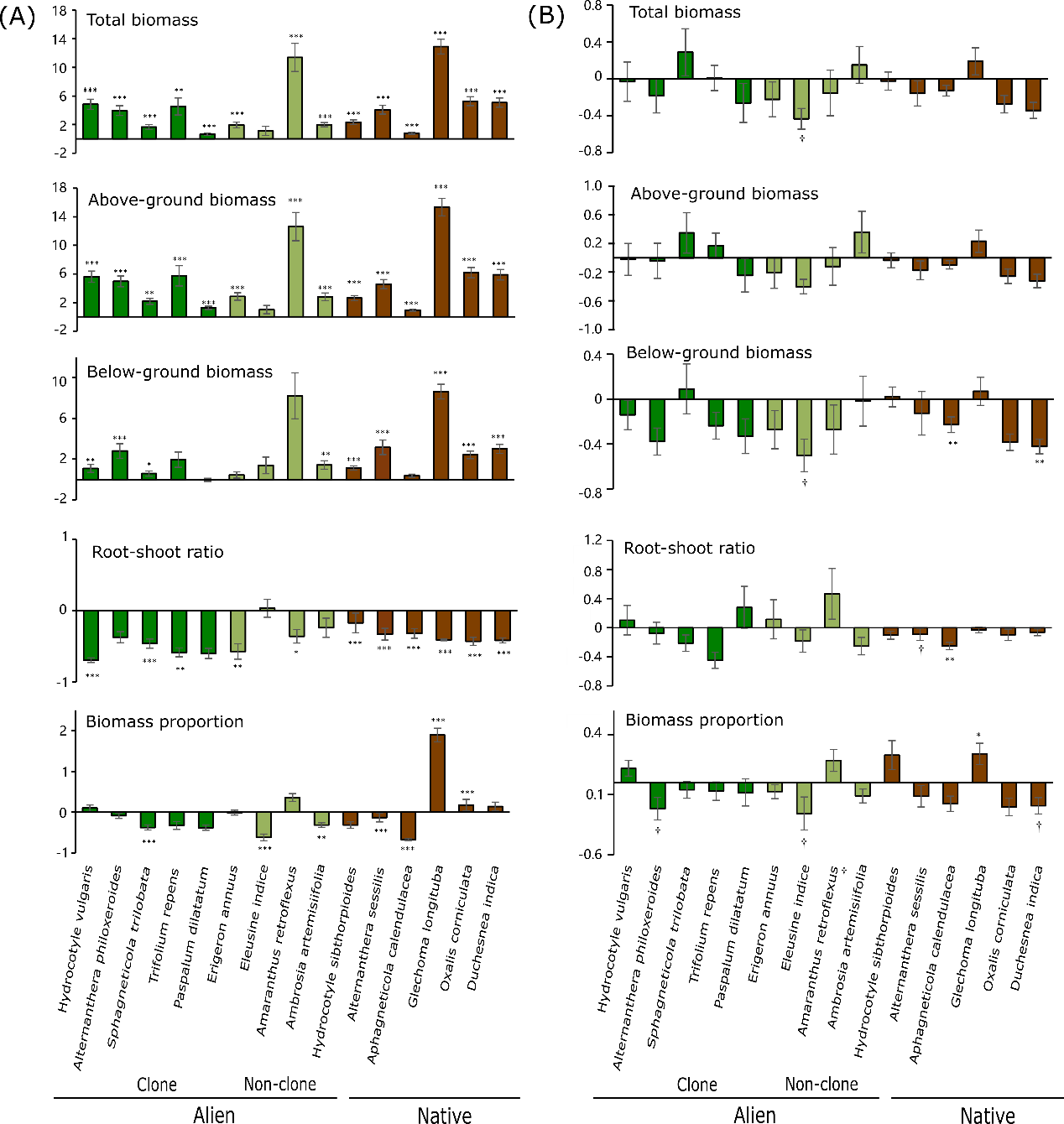


# Fig. S2 Relative enemy suppression responses for total biomass, above-ground biomass, and biomass proportion for each native and alien target species under without nutrient addition. Bars represent means ±SE. Asterisks above bars for each species indicate a significant enemy suppression and nutrient addition response from t-tests comparing trait values: *, 0.05＞*P* ＞0.1; **, *P* < 0.05; ***, *P* < 0.01.


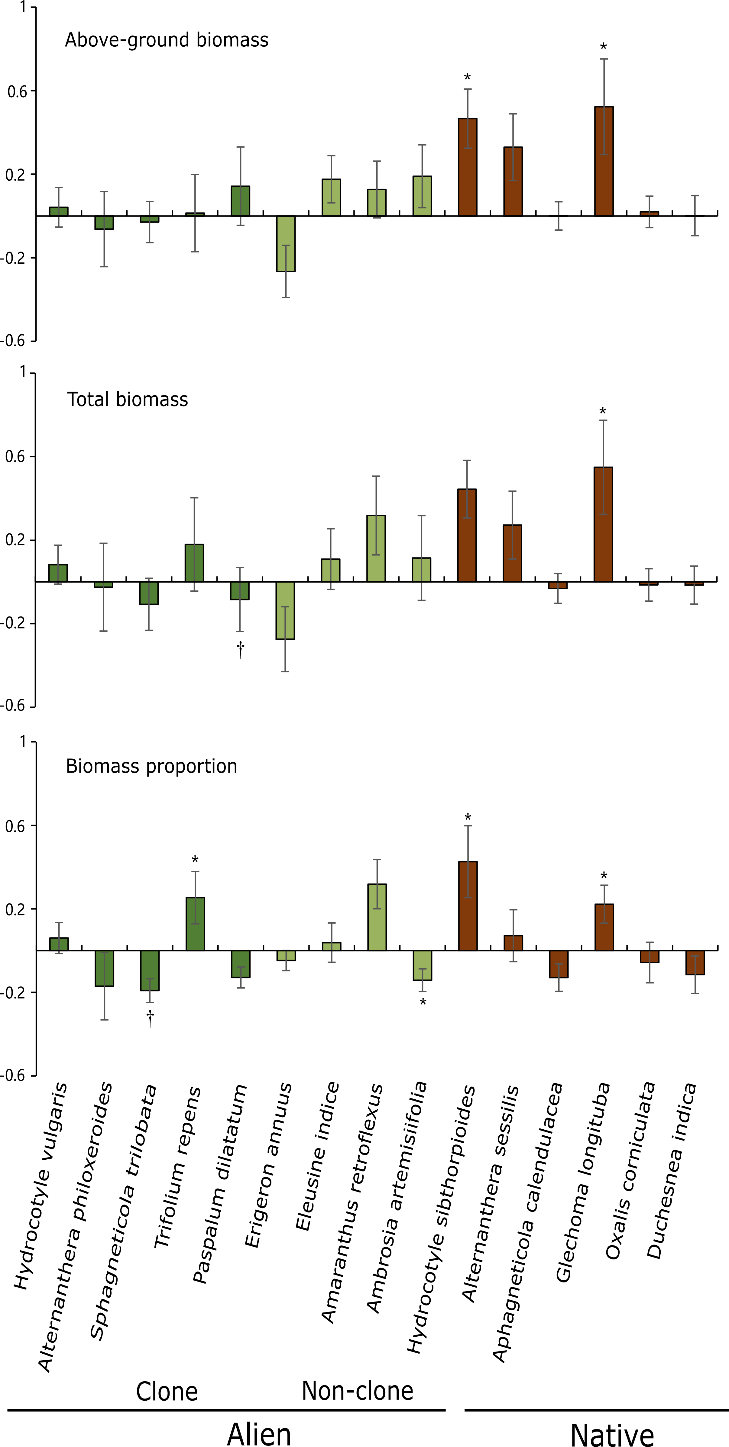


# Fig. S3 Effects of nutrient addition (without vs. with), enemy suppression (with vs. without) and their interaction on below-ground biomass for alien target species and native community. +ES=with enemy suppression, -ES=without enemy suppression, +N=with nutrient addition, -N=without nutrient addition. Significance levels: *P < 0.05* are indicated with asterisks (^∗^), *0.05 < P < 0.1* are indicated with daggers (^†^), *P > 0.1* are indicated with ‘ns’.


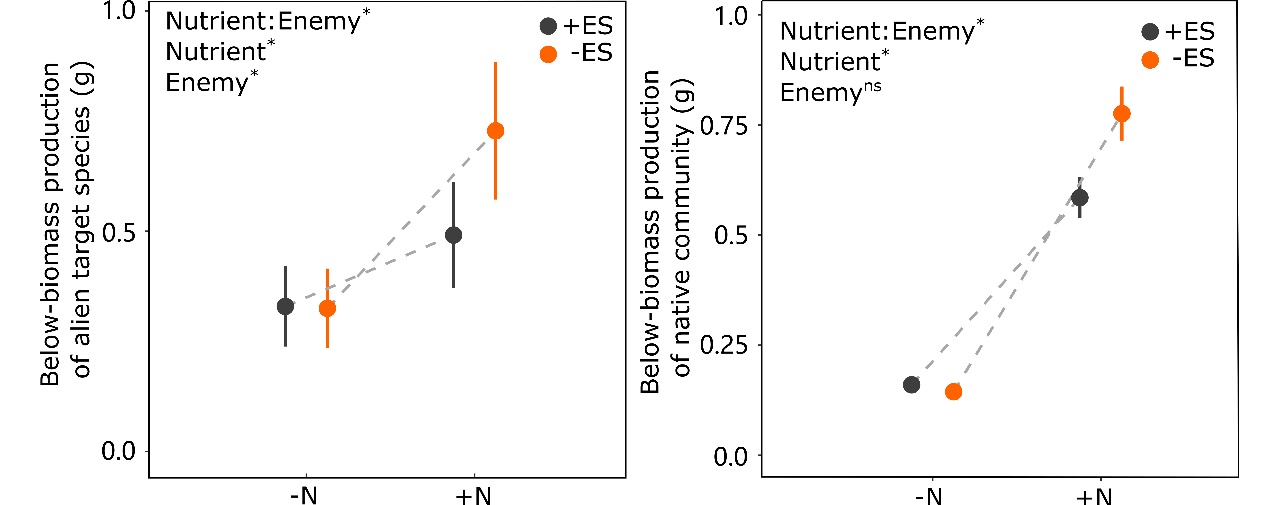


# Fig. S4 Graphical illustration of the experimental design. The panel shows an overview of the nutrient addition treatment, enemy suppression treatment and the locations of the alien target species and native community plants in the pots.


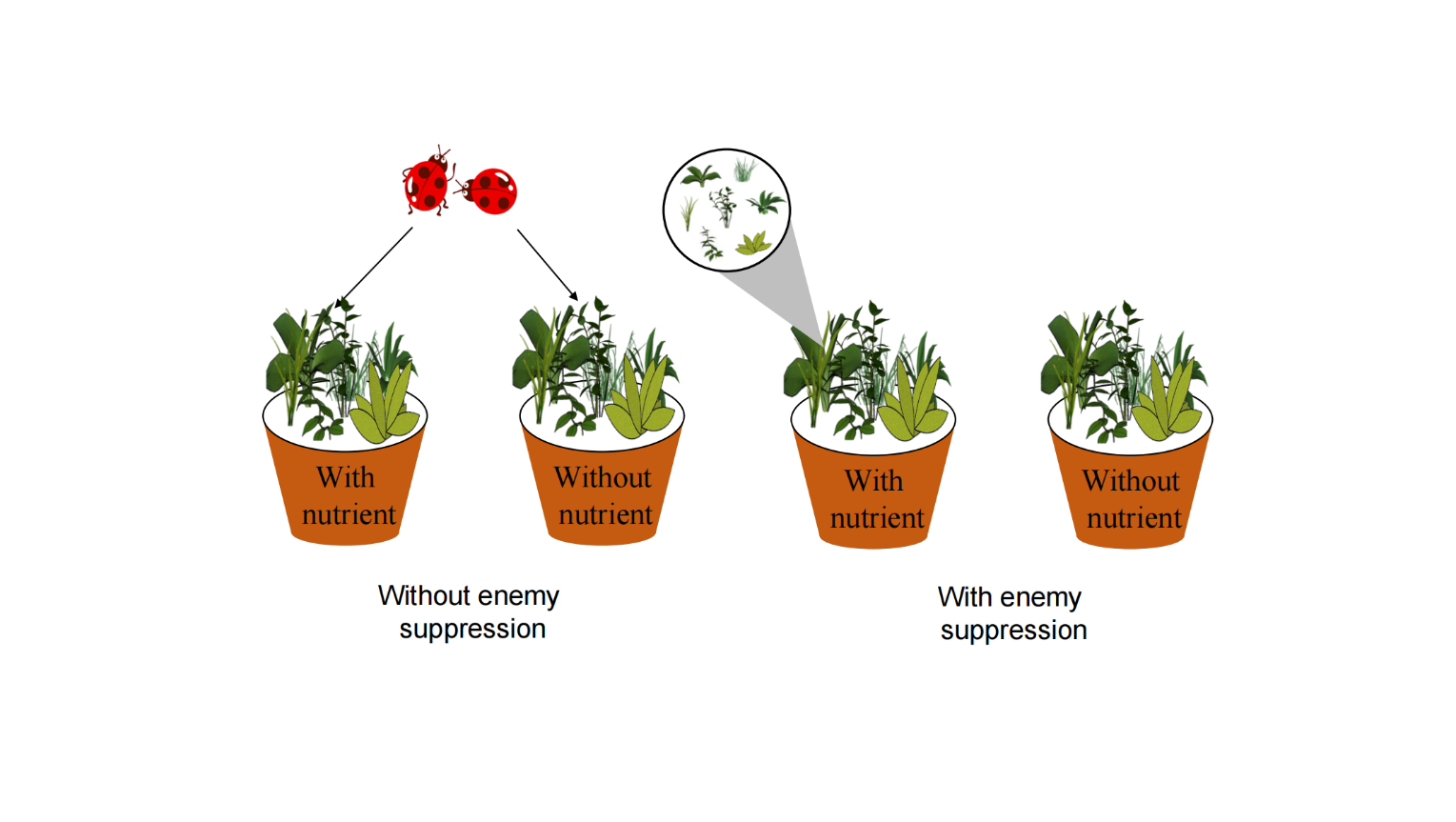

Supplement: Supplementary file 1 — Data S1 [file ECE3-14-e10853-s001.docx]
